# Chronic prosthetic joint infections with a draining sinus. Who should receive suppressive antibiotic treatment?

**DOI:** 10.5194/jbji-6-43-2020

**Published:** 2020-10-30

**Authors:** Karel-Jan Lensen, Rosa Escudero-Sanchez, Javier Cobo, Alex Soriano, Marjan Wouthuyzen-Bakker

**Affiliations:** 1 Department of Infectious Diseases, University Medical Center Groningen, University of Groningen, Groningen, the Netherlands; 2 Department of Infectious Diseases, Hospital Ramón y Cajal, IRYCIS, Madrid, Spain; 3 Department of Infectious Diseases, Hospital Clinic, Barcelona, Spain; 4 Department of Medical Microbiology and Infection Prevention, University Medical Center Groningen, University of Groningen, Groningen, the Netherlands

## Abstract

The benefit of suppressive antibiotic treatment in inoperable patients with a chronic periprosthetic joint infection and a sinus tract is unknown. Some physicians prefer to just let the sinus drain, while others prefer antibiotic treatment. In this viewpoint article we discuss the advantages and disadvantages of suppressive antibiotic treatment in this particular patient group.

## 1 Viewpoint

The surgical management of prosthetic joint infections (PJIs) consists of extensive irrigation and debridement in acute infections and prosthesis exchange in chronic infections (Osmon et al., 2013). However, surgical management may not always be an option: e.g., patients may not be eligible or fit for surgery, surgery is not expected to result in a significant functional improvement, or patients may decline surgery themselves. In these cases, the goal of treatment shifts towards suppression of the infection to reduce symptoms and to delay progression of the infection. This may be achieved by prescribing lifelong suppressive antibiotic treatment (SAT).

The efficacy of SAT reported in the literature differs, ranging from 50 to 72 % (Segreti et al., 1998; Rao et al., 2003; Wouthuyzen-Bakker et al., 2016). The described clinical outcome largely depends on the studied population, the indications to prescribe SAT, and the definitions used to define treatment success. The largest, recently published, retrospective multicenter cohort study by Escudero et al. (2020) evaluated 302 patients with SAT. The authors demonstrated that the efficacy of SAT was around 60 % in patients with a minimal follow-up of 6 months (Escudero et al., 2020). In this study, treatment failure was defined as the appearance or persistence of a sinus tract, the need for debridement or replacement of the prosthesis due to persistence of the infection, or the presence of uncontrolled symptoms. Acute as well as chronic PJI patients without potentially curative surgical treatment were included.

**Table 1:** Summary of questionnaire on prescribing SAT in patients with a chronic PJI and a sinus tract. Sent to medical specialists (orthopedic surgeons (n=15), infectious disease specialists (n=30), and medical microbiologists – n=3).

	Yes (%)	No (%)	Sometimes (%)
Do you prescribe SAT${}^{1}$ to patients with a PJI and a sinus tract when the patient is inoperable?	40 %	10 %	50 %
Goal of therapy			
Avoid bacteremia Avoid/reduce degree of anemia Reduce inflammation Reduce pain Other${}^{2}$	40 % 8 % 58 % 48 % 23 %		
How do you determine the type of SAT?			
Based on a culture of the sinus tract Based on a culture of synovial fluid Empirically Not applicable${}^{3}$ Other${}^{4}$	6 % 78 % 0 % 6 % 10 %		
Do you administer IV antibiotic first before switching to oral SAT?	15 %	29 %	56 %
Do you include rifampin in SAT?${}^{5}$	26 %	74 %	
When the sinus tract remains open during SAT, what do you do?			
Maintain the same SAT Broaden the SAT Stop the SAT Obtain new cultures Not applicable${}^{3}$	29 % 0 % 27 % 33 % 11 %		

${}^{1}$ SAT: suppressive antibiotic therapy. ${}^{2}$ For example, patients struggling with sinus output, to stop drainage. ${}^{3}$ Not prescribing SAT to patients with a sinus tract. ${}^{4}$ Combination of all options, based on cultures obtained during debridement. ${}^{5}$ In case of a susceptible microorganism.

Whether the efficacy of SAT is comparable between different patient groups is not clear. In the study of Escudero et al. (2020), treatment failure was associated with an age below 70 years, a causative microorganism other than Gram-positive cocci, and location of the prosthesis in the upper limb. Interestingly, there were 133 cases with a sinus tract, and the presence of a sinus tract was not associated with a higher failure rate. This finding is somewhat surprising, as the presence of a sinus tract suggests a high bacterial burden, and therefore it is reasonable to assume that this would be associated with a worse clinical outcome. However, external drainage and achieving a natural load reduction of bacteria via the sinus could theoretically be sufficient to control the infection without the need for additional antibiotic treatment. This would advocate withholding SAT in patients with a draining sinus, especially since adverse events and the selection of antibiotic resistance should be avoided as much as possible. The most reported side effects of SAT are gastro-intestinal complaints (e.g., nausea and abdominal pain) and skin problems, in particular in the case of tetracycline usage (phototoxicity). These symptoms are most prominent in elderly patients, which is the population in whom SAT is mostly described. In the study of Escudero et al., the development of antibiotic resistance has been described in 23 % of patients receiving SAT with documented microbiological failure (Escudero et al., 2020). Other hazards of SAT consist of interaction with other drugs and the selection of other microorganisms in the infected area or distant locations, including *Clostridioides difficile*, with potentially harmful consequences for the patient (Lau et al., 2018). On the other hand, withholding SAT may theoretically result in progressive loosening of the prosthetic joint with subsequent worsening of pain. In addition, opening of a new sinus or worsening of the existing sinus may be perceived as mutilating for patients. Moreover, chronic inflammation may lead to chronic fatigue, anemia, and – less frequently – local squamous carcinoma or AA amyloidosis (Visuri et al., 2016; Zhang et al., 2019). In this regard, withholding SAT may reduce the quality of life of patients. Currently, there are no data supporting the prescription of SAT in patients with a sinus tract, nor do data exist to support to withhold it.

In a short explorative questionnaire sent to orthopedic surgeons, infectious disease specialists, and medical microbiologists involved in the treatment of PJI, they were asked whether and/or how they manage PJI patients with sinus tracts not eligible for curative surgery, regardless of the reason. Results of this questionnaire are presented in Table 1. Most of the respondents would initiate or at least consider SAT in patients with a sinus tract. The primary goals of therapy were to reduce inflammation, to reduce pain, and to avoid bacteremia. The majority would tailor SAT based on synovial fluid cultures and prefer to reduce the bacterial load first by giving an induction treatment with IV antibiotics. Some physicians even prefer to add rifampin to the SAT regimen in case of susceptible organisms. In case the sinus tract remains open despite SAT, physicians choose to either maintain SAT, stop SAT, or reevaluate its efficacy by obtaining new cultures. Overall, the results of this explorative survey demonstrate that there is considerable variability in how patients with a sinus tract are managed.

Due to the current lack of evidence favoring one particular approach, a clinical study is initiated within the ESCMID Study Group on Implant Associated Infections (ESGIAI). It this multicenter study, chronic PJI patients with a sinus tract not eligible for surgery will be evaluated, and the clinical outcome of patients who received SAT will be compared to those in whom SAT was withheld. The main outcome parameters will be symptom control, the occurrence of bacteremia, and the need for surgical intervention to control the infection. For those patients who received SAT, special attention to clinical outcome and the induction of resistance will be given to those who received rifampin as part of the SAT regimen. The aim of this study is to provide future guidance on how to manage these patients.

In conclusion, prescribing SAT to patients with a chronic PJI and a sinus tract not eligible for surgery has clinical advantages and disadvantages. At the moment, there is no consensus on how to manage these patients, and treatment decisions are currently based on an individual basis. Future studies are needed to determine the best treatment strategy in this particular patient category.

## Data Availability

No data sets were used in this article.
